# Matrix Matters: Differences of Grand Skink Metapopulation Parameters in Native Tussock Grasslands and Exotic Pasture Grasslands

**DOI:** 10.1371/journal.pone.0076076

**Published:** 2013-10-02

**Authors:** Konstanze Gebauer, Katharine J. M. Dickinson, Peter A. Whigham, Philip J. Seddon

**Affiliations:** 1 Department of Botany, University of Otago, Dunedin, New Zealand; 2 Department of Information Science, University of Otago, Dunedin, New Zealand; 3 Department of Zoology, University of Otago, Dunedin, New Zealand; The Australian National University, Australia

## Abstract

Modelling metapopulation dynamics is a potentially very powerful tool for conservation biologists. In recent years, scientists have broadened the range of variables incorporated into metapopulation modelling from using almost exclusively habitat patch size and isolation, to the inclusion of attributes of the matrix and habitat patch quality. We investigated the influence of habitat patch and matrix characteristics on the metapopulation parameters of a highly endangered lizard species, the New Zealand endemic grand skink (*Oligosoma grande*) taking into account incomplete detectability. The predictive ability of the developed zxmetapopulation model was assessed through cross-validation of the data and with an independent data-set. Grand skinks occur on scattered rock-outcrops surrounded by indigenous tussock (bunch) and pasture grasslands therefore implying a metapopulation structure. We found that the type of matrix surrounding the habitat patch was equally as important as the size of habitat patch for estimating occupancy, colonisation and extinction probabilities. Additionally, the type of matrix was more important than the physical distance between habitat patches for colonisation probabilities. Detection probability differed between habitat patches in the two matrix types and between habitat patches with different attributes such as habitat patch composition and abundance of vegetation on the outcrop. The developed metapopulation models can now be used for management decisions on area protection, monitoring, and the selection of translocation sites for the grand skink. Our study showed that it is important to incorporate not only habitat patch size and distance between habitat patches, but also those matrix type and habitat patch attributes which are vital in the ecology of the target species.

## Introduction

The concept of metapopulations has gained considerable traction in conservation biology since an increasing number of animal and plant populations now inhabit fragmented landscapes, often resulting from habitat degradation and destruction [1–3]. Metapopulations consist of a number of small populations that occur in spatially distinct habitat patches surrounded by a landscape that is generally unsuitable for species occupancy (termed ‘matrix’ [4,5]). Individual sub-populations resident in habitat patches embedded in the matrix may become extinct but migrating individuals may be able to (re-)colonise unoccupied habitat patches [4,6]. The metapopulation framework introduces a spatial dimension to modelling population dynamics, connecting individual populations which are spatially distributed in a wider landscape [7,8]. Conservation biologists and managers are thus able to investigate metapopulation responses to ecological processes at the landscape scale, the level of connectivity of individual populations, and their probabilities of extinction or establishment.

To develop successful species conservation strategies information on the influence of habitat patch characteristics on metapopulation parameters are needed. According to the “area and isolation” paradigm developed by Hanski [9], and supported by empirical and theoretical research, the extinction rate of a habitat patch is inversely related to its size [10,11], whereas the colonisation probability is inversely related to the degree of isolation of the habitat patch [12–14]. Although the discrimination of habitat patches of different size and isolation has been factored into metapopulation models in the past, more realistic models incorporating the quality of habitat patches have been called for [15–17]. Moilanen and Hanski [12] accounted for habitat quality by adjusting the effective habitat patch size but found the quality of the model did not improve with inclusion of the chosen environmental variables. On the other hand, research by Dennis and Eales [16] and Thomas et al. [17] showed that habitat quality strongly determined butterfly occupancy probabilities in fragmented landscapes. Similarly, Jaquiery et al. [18] demonstrated that habitat quality significantly influenced occupancy probabilities for greater white-toothed shrew (*Crocidura russula*) metapopulations, although the extinction probabilities were not lowered as expected.

Early metapopulation research treated the matrix surrounding the habitat patches as uniform and separate from habitat patches. However, recent research shows that the matrix is heterogeneous and can affect patch populations [19] and migration [20,21]. Although research in landscape ecology has demonstrated that matrix composition can influence the speed, distance and direction of dispersing animals, rarely are these factors incorporated into metapopulation models or the estimation of colonisation and extinction probabilities [21–25]. Thus, it is important to know to what extent different habitat patch and matrix characteristics can influence the different parameters of metapopulations in order to understand or to predict changes in the overall metapopulation dynamics.

Several approaches have been developed to model metapopulation parameters, ranging from models based on the occupancy state of habitat patches (a species’ presence or absence, i.e. occupancy models), to individual-based simulations that model the dynamics of each local population [26,27]. An advantage of occupancy models is the rapidity of collecting presence-absence data over large areas. In this approach the detection probability of a species has to be explicitly modelled to avoid the bias of false-absences [28–30]. Moilanen [31] and Tyre et al. [32] showed that incomplete detection of a species can result in biased estimation of metapopulation parameters and can reduce the predictive abilities of metapopulation models. Furthermore, to be able to consider model results in species management the predictive abilities of the models need to be evaluated [33,34].

Our study takes into account the incomplete detectability of a species in the evaluation of the effects of habitat patch and matrix characteristics on metapopulation parameters. Our goal was to quantify the degree to which habitat patch and matrix characteristics influence the underlying dynamics of a metapopulation of the highly endangered, New Zealand grand skink (*Oligosoma grande*). Presence-absence data were used to create models identifying the relative importance of habitat patch characteristics on metapopulation parameters. We investigated what effect the type of matrix between habitat patches, habitat patch size and composition, isolation and vegetation cover have on patch-specific colonisation, extinction, occupancy and detectability probabilities for the grand skink metapopulation. Additionally, we measured the predictive ability of the final model to provide guidance for conservation managers who, for example, might use the models to assess the quality of new conservation land.

## Methods

### Ethics statement

All work was approved by the University of Otago Animal Ethics committee (protocol number 103/08) and the Department of Conservation (National Permit Number: OT-25159-FAU). The study was undertaken with permission of the owners of the accessed farmland.

#### 2.1: Study species

The grand skink is an endangered, viviparous skink that occupies schist-rock outcrops scattered throughout the native tussock and exotic pasture grasslands of the central South Island of New Zealand. Today, grand skinks occur in only eight percent of their historic range [35]. Introduced mammalian predators and the loss of habitat have been identified as their major threats [35]. The omnivorous grand skinks feed on insects and fruits that they mostly find on their residential outcrop [35,36]. Outcrops can be inhabited by varying numbers of grand skinks. Even on relatively small outcrops populations of up to 20 grand skinks can be found, while large outcrop populations can exceed 50 individuals ([37,38], KG, pers. obs.). Grand skinks show strong site fidelity and have only occasionally been observed in vegetation away from a rock outcrop [39,40]. Furthermore, detailed tracking studies found no evidence that adult grand skinks use the grasslands around outcrops in pasture or in tussock habitats (KG, unpublished data). The grassland matrix surrounding the outcrops is used primarily during dispersal of a small percentage of young animals while adult animals spend most of their life on the same outcrop [37,39,41].

#### 2.2: Study area

**Figure 1:**
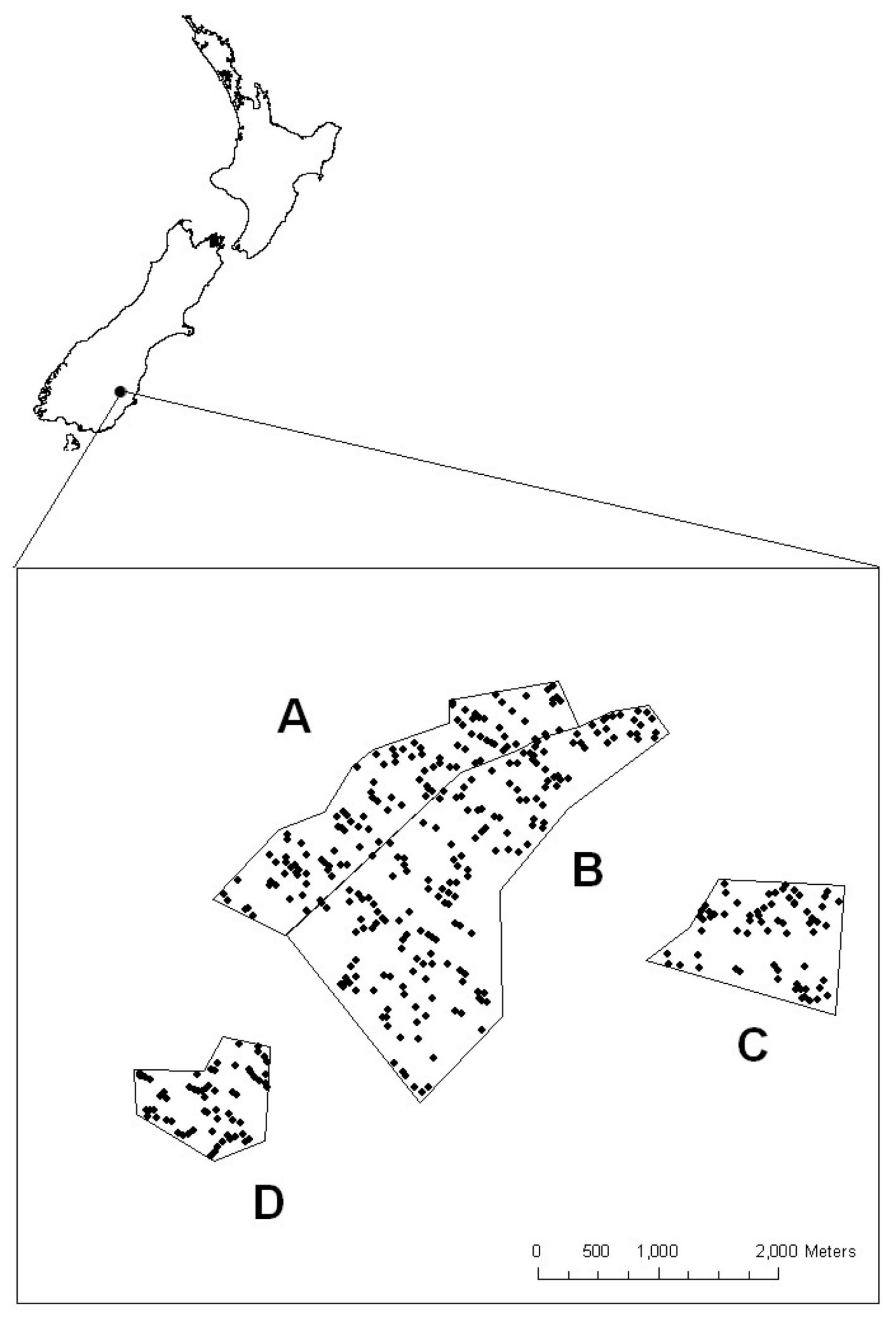
Location of study sites with all monitored habitat patches (black marks). The 2006-08 data was collected in study sites A and B comprising of exotic pasture grasslands and indigenous tussock grasslands, respectively. Study site C (tussock grasslands) and D (pasture grasslands) were used for collection of evaluation data in 2010.

The study was conducted at Macraes Flat (45°28′ S, 170°28′ E; Figure 1), a population stronghold for grand skinks, monitored and managed by New Zealand’s Department of Conservation for over two decades. Before first human settlement in the 13^th^ century, extensive indigenous tall tussock (*Chionochloa* spp.) grasslands up to 1.5 m high dominated the area. Furthermore, New Zealand was lacking terrestrial mammalian predators. Over the intervening period, after the Maori settlement in the 13^th^ century and increasingly after European settlement 170years ago, the indigenous grasslands have been modified to varying degrees by grazing and burning, through the application of fertiliser and over-sowing with preferred exotic forage plant species, e.g. *Lolium* spp. and *Trifolium repens* [42–44]. Today, land use for the most part comprises intensive cattle and sheep grazing. Additionally, introduced mammalian predators occur throughout the area. However, within the conservation reserve at Macraes Flat the New Zealand Department of Conservation is protecting remnant indigenous tussock grassland ecosystems. At the study site a property boundary divided protected tussock grasslands to the south-west from pasture grasslands on private farmlands to the north-east.

#### 2.3: Data collection

Occupancy data were collected by surveying all outcrops in pasture (n= 115) and in tussock (n= 174) grasslands at the study site (Figure 1, Site A and B, respectively). An outcrop was classified as a schist-rock tor or group of schist-rock tors separated by a minimum of 10 m of matrix grassland from any other schist-rock tor [37,45]. For the purposes of this paper, these outcrops are referred to as habitat patches. Each habitat patch was surveyed annually over three years (2006-2008) with four surveys within a two-week time period in January (mid-summer) each year. Each habitat patch was searched until at least one grand skink was sighted or 5 minutes had elapsed without a sighting, using a standard search protocol modified from Roughton and Seddon [46]. A habitat patch was recorded as having skinks present (coded 1) when at least one skink was sighted, and as absent (coded 0) when no skink was sighted within the 5 minute period. Each survey was undertaken in sunny, warm and low wind weather conditions when skinks are most likely to bask and forage [40,47] providing highest detection probabilities. During each set of four surveys observers were rotated so they would not monitor the same habitat patch twice. Similar weather conditions for each survey day and the rotation of observers was assumed to provide detection probabilities independent of survey specific factors.

We identified five habitat patch characteristics that can be rapidly collected during surveys, and which were likely to be important for the ecology of grand skinks: patch size, composition, vegetation cover, isolation and surrounding matrix. Habitat patch size has been shown to be an important attribute through its assumed correlation with population size which in turn decreases the risk of extinction by stochastic events and therefore increases occupancy probability [2]. In our study, habitat patches were categorised according to their relative size into three broad size groups: small, medium and large.

The composition of a habitat patch was categorised as habitat patches consisting of either one distinct rock-tor (discrete) or a group of rock-tors (clustered). A cluster of rock-tors within a habitat patch may provide more crevices of varying sizes, potentially providing more refugia for grand skinks [41]. Cracks and crevices are also spaces for invertebrates which form a large part of the grand skink’s diet. On the other hand, distinct rock-tors may be more likely to provide crevices with enough depth to protect grand skinks from weather extremes.

For the purpose of this study vegetation cover was categorised on each habitat patch as one of three categories: none, moderate and abundant. Vegetation on a rock-tor provides food in form of fruits and serves as an attractant and habitat for invertebrates, which are the major part of the grand skink diet. Additionally, vegetation can act as refugia for grand skinks during predatory attacks as well as shade during hot temperatures allowing for optimal thermoregulation. The catgorisation into three broad groups allowed for rapid data collection over a large area and required less training of observers.

The matrix type surrounding a habitat patch may influence invertebrate composition, abundance [36], and also movements of grand skinks between habitat patches [37,38]. Therefore habitat patches were classified as being surrounded by either pasture grasslands or tussock grasslands.

Previous studies found that movements of grand skinks were predominantly less than 50 m, with only occasional movements of over 400 m [40,48]. Therefore, the distance between habitat patches could significantly influence colonisation and occupancy probabilities. A large number of connectivity measures have been suggested in the literature [7,49]. One of the simplest measures is the distance to the nearest neighbour habitat patch. This measure is often described as too simplistic, and Prugh [49] reported that the distance to the nearest occupied habitat patch would perform significantly better as a predictor for species occupancy. However, because grand skinks have a low detectability [45], recorded absences could include presences of grand skinks (false-absences) introducing an error into connectivity measures based on occupancy data. Therefore we used the Euclidean distance from each habitat patch to the nearest neighbouring patch as a measure of isolation of habitat patches in this study. The distances were measured using aerial photographs and ArcView 10 GIS software.

#### 2.4: Statistical analysis

The metapopulation parameters, occupancy probability, colonisation probability and extinction probability were simultaneously estimated using logistic regression models that correct for incomplete detectability using a second logistic regression-based detectability model in the computer program PRESENCE 3.0 (for details see [50,51]). According to our sampling design we used the multiple season analysis to estimate occupancy, colonisation, extinction and detection probabilities [28]. For each habitat patch a survey history can be created using the presence or absence of grand skinks recorded during each of the four surveys per year. This information was used to estimate the probability of detection for each habitat patch dependent on the patch-specific characteristics. The patch-specific probability of colonisation, extinction and occupancy is then estimated for each habitat patch [28,51,52].

One aim of this study was to investigate the relative importance of habitat patch characteristics on the metapopulation parameters. Since all parameters are estimated simultaneously in the models created in PRESENCE, including all biologically meaningful combinations of habitat characteristics for each metapopulation parameter would result in a large number of candidate models. Therefore, we employed a selection process to identify the most relevant habitat characteristics for each metapopulation parameter separately, as suggested by MacKenzie [53]. While creating a set of models investigating the relative importance of habitat patch characteristics for one metapopulation parameter, we used intercept only models (null-models) for the other three parameters [53]. To rank the relative importance of the habitat patch characteristics a model set was created for each of the four metapopulation parameters representing all combinations of the habitat patch characteristics to ensure each were represented equally in the model sets [54]. Within each model set, models were ranked using values of the second-order bias corrected Akaike information criterion (AICc [55]). To identify the relative importance of a particular habitat patch characteristic the Akaike weights derived from of all models that included the focal attribute were added [54].

The relative importance of the habitat patch characteristics size, composition, vegetation cover and matrix type were investigated for all metapopulation parameters, whereas habitat patch isolation was included only in the model sets estimating colonisation probability and occupancy probability. Although it has been shown that isolation influences extinction probabilities in other species (e.g. via the rescue effect [12,56]), we could not include this variable in out model-set for extinction probability because the low number of recorded extinction events did not allow the models to converge. We can therefore draw no conclusion about the effect of isolation on extinction probabilities in this study.

Candidate model-sets for each parameter included all combinations of the patch characteristics, resulting in 31 models for occupancy and colonisation probability, and 15 models for extinction and detection probabilities (Appendix S1). Habitat patch characteristics which had summed AIC weights of >0.5 for a metapopulation parameter were used to create models for the candidate set investigating temporal variation in the metapopulation parameters. This candidate set included the following models: (1) all parameters were allowed to vary between years, (2) only colonisation probabilities remained constant, all other parameters varied between years, (3) only extinction probabilities remained constant, (4) only detection probabilities remained constant and (5) colonisation and extinction probabilities remained constant. We allowed detection probability to vary between years in all models acknowledging the potential differences in weather conditions and observer experience between years.

#### 2.5: Evaluation

A further aim of this study was to evaluate the predictive abilities of our top-ranked model to provide guidance for conservation biologists. We used two evaluation measures, the threshold dependent true skill statistic (TSS) developed by Allouche et al. (2006), and the threshold independent area under the curve (AUC) of the receiver operating characteristic (ROC) function [33].

The TSS is unaffected by prevalence or the size of the validation set, taking into account sensitivity (correctly identified presences) and specificity (correctly identified absences), and ranges between -1 (performance no better than random) to +1 (perfect classification [57]). To convert the continuous probabilities predicted by the logistic regression model into dichotomous presence-absence data we used an optimization threshold which was determined as the cross-point of sensitivity and specificity plotted against a number of thresholds [58,59]. The obtained presences and absences were then compared to the validation data-set by determining sensitivity, specificity and correct classification rates ranging from 0 (no correct classification) to 1 (perfect classification [33]).

The AUC does not directly identify a classification rule for converting probabilities into presences and absences [33]. It is obtained by plotting 1-specificity versus sensitivity for all thresholds with AUC values varying from 0.5 for an indiscriminate model to 1.0 for a perfect model [60,61].

To evaluate the predictive abilities of the highest ranked model from our previous analysis we used 10-fold cross-validation (internal evaluation) and independent data (external validation). The 10-fold cross-validation procedure randomly splits the data into ten independent groups, using nine groups to train the model and the 10^th^ group to validate the model. The training and validation process was repeated ten times enabling the calculation of the standard deviation and variance for the AUC and TSS of the highest ranked model [61,62].

Using the habitat patch characteristics in the validation data-set the individual probability of occupancy for each habitat patch for the first year was estimated using the logistic regression model for occupancy probability of the top-ranked model. The occupancy probabilities for the following two years are estimated by calculating predicted extinction (ε) and colonisation (γ) probabilities for each habitat patch with the logistic regression models obtained in PRESENCE and deriving the occupancy probabilities (ψ _t+1_) from the occupancy probabilities (ψ _t_) of the previous year:

$$\psi_{t+1}=\psi_{t}*\;(1-\varepsilon_{t})\;+\;(1-\psi_{t})\;*\gamma_{t}$$

[50]

To test the transferability of the predictive accuracy of a model to other areas, testing data should be spatially independent from training data [63,64]. To assess how well our occupancy model predicted skink occurrences in other areas we collected habitat patch characteristics and determined the presence or absence of grand skinks for 64 habitat patches surrounded by pasture and 67 habitat patches surrounded by tussock grasslands during four surveys in December 2010 (Figure 1 Sites D and C, respectively). The final model was trained with all available data from the years 2006-2008 and the optimized threshold identified. The data collected on habitat patch characteristics in 2010 were used to predict occupancy of grand skinks in the two new study sites and the model’s predictive abilities measured using TSS and AUC with combined presence-absence data of the four surveys from 2010.

AUC and TSS were calculated using software R software version 2.12.0 [65,66] with the ‘verification’ package [67]. All mean values are reported with +/- standard errors in the result section.

## Results

Combining all four surveys of each year and collating the proportion of patches where skinks were recorded as being present resulted in a naïve estimated occupancy rate for the whole study site of 0.42, 0.48 and 0.47 in 2006, 2007 and 2008, respectively. We recorded 33 colonisation and 19 extinction events between 2006 and 2007, and 34 colonisations and 35 extinctions between 2007 and 2008.

### 3.1: Habitat and matrix characteristics

Pairwise comparisons were used to identify possible correlations between habitat patch characteristics. Three pairs showed low correlations: size and vegetation (r = 0.21), size and composition (r = 0.13) and habitat and vegetation (r = 0.12). All other pairwise comparisons showed no significant correlation.

After ranking the habitat patch characteristics according to their relative importance (AIC weights) for each metapopulation parameter, the occupancy probability model retained (in order of relative importance) the variables size and matrix type; the colonisation probability model retained size and matrix type; the extinction probability model retained size, composition, vegetation cover and matrix type, and the detection probability model retained size, composition and matrix type (Table 1).

**Table 1:** Summed Akaike weights for models including a particular variable.

	Metapopulation Parameter			
Habitat patch characteristic (possible values)	Occupancy probability	Colonisation probability	Extinction probability	Detection probability
Size (small, medium or large)	1.00*	1.00*	0.97*	1.00*
Composition (clustered or discrete)	0.49	0.29	0.97*	1.00*
Vegetation (none, moderate or abundant)	0.28	0.33	0.69*	0.44
Isolation (distance) (mean = 45.5 m, SE = 1.4 m, range: 10.0 m-174.8 m)	0.40	0.27	-	-
Matrix (pasture or tussock)	1.00*	1.00*	0.57*	0.98*

* Variables included in the overall model for the corresponding parameter

**Figure 2:**
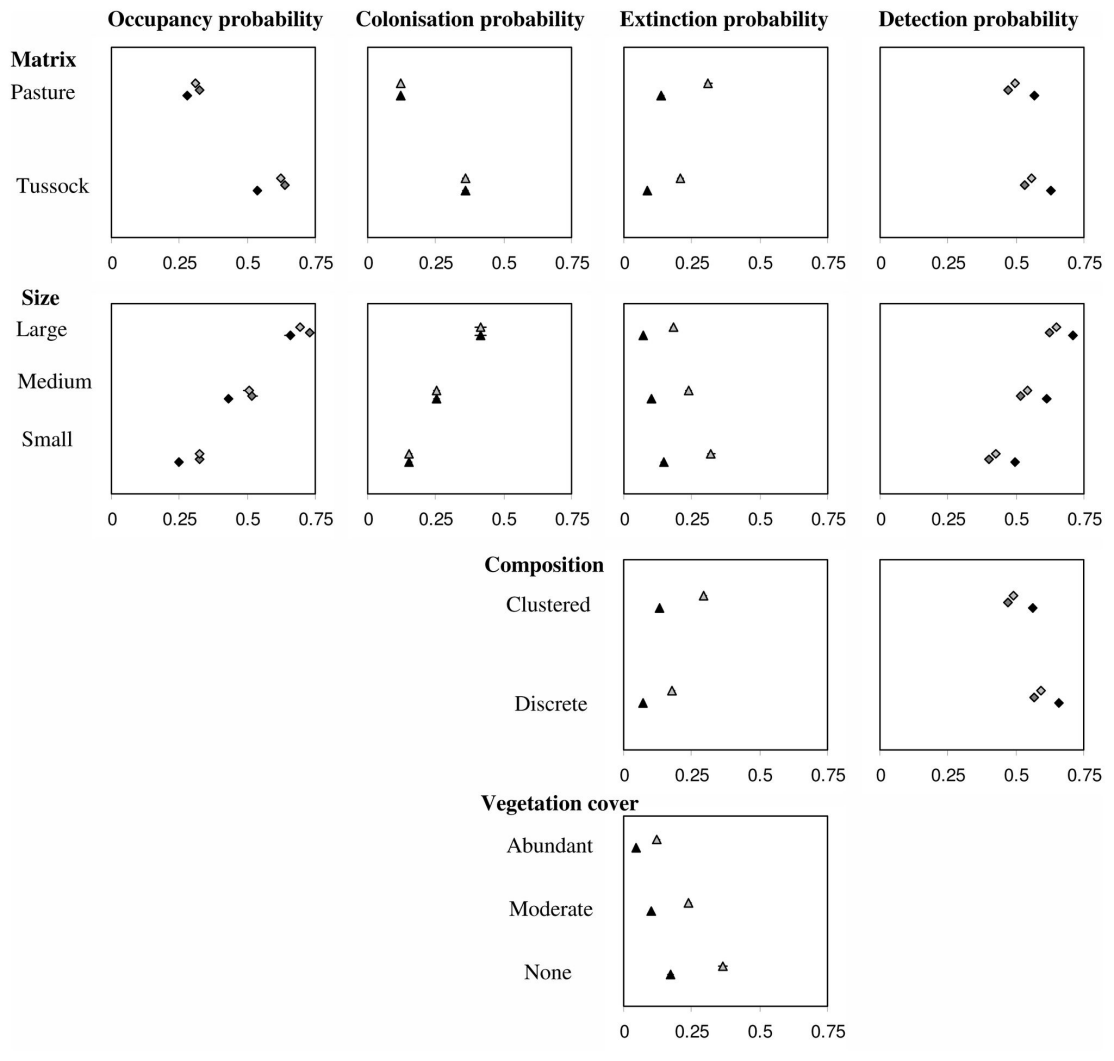
Influence of habitat patch characteristics on the mean estimated occupancy, colonisation, extinction and detection probability. Displayed are habitat patch characteristics which were included in the final model for each metapopulation parameter. Occupancy and detection probabilities were estimated for 2006 (black circles), 2007 (dark gray circles) and 2008 (light gray circles). Colonisation and extinction probabilities were estimated from occupancy differences between 2006 and 2007 (black triangles), and 2007 and 2008 (gray triangles).

Overall, size of the habitat patch was the highest ranked variable for each of the metapopulation parameters, closely followed in importance by matrix type. Larger habitat patches had higher occupancy, colonisation and detection probabilities and a lower extinction probability (Figure 2). Matrix type had similar combined model weights compared to habitat patch size for occupancy, colonisation and detection probabilities. Matrix type was ranked lowest for extinction probabilities but still accounted for combined model weights of 0.57 and therefore was included in the final model (Table 1). Habitat patches had higher occupancy, colonisation and detection probabilities and lower extinction probabilities if surrounded by tussock grasslands compared to pasture grasslands (Figure 2). The composition of habitat patches was included only in the models for extinction and detection probabilities (Table 1); habitat patches consisting of a cluster of outcrops had higher extinction probabilities and lower detection probabilities than did habitat patches with one distinct outcrop (Figure 2). High-ranked models for extinction probabilities also included vegetation cover (Table 1), with greater vegetation cover associated with decreased extinction probabilities (Figure 2). Distance to the nearest habitat patch (mean = 45.5m +/- 1.4m) yielded only very low combined model weights and therefore was not included in the occupancy and colonisation models (Table 1).

Using all retained variables for each metapopulation parameter, the most parsimonious model describing variation in metapopulation parameters over time kept the colonisation probability constant but varied extinction and detection probabilities between years. The other models received little support with delta AICc >2.00 (Table 2). In the most parsimonious model, the average extinction probability increased in the period 2007/8 to 0.25 (+/- 0.12), compared to a mean extinction probability of 0.11 (+/- 0.07) in 2006/7. Average occupancy probabilities were lowest in 2006 at 0.43 (+/- 0.21), but increased to 0.51 (+/- 0.22) and 0.50 (+/- 0.22) in 2007 and 2008, respectively. The top-ranked model kept colonisation probability constant at 0.27 (+/- 016) over the three year period. Detection probabilities were highest in 2006 with 0.60 (+/- 0.11) and decreased to 0.51 (+/- 0.11) in 2007 and 0.53 (+/- 0.10) in 2008 (Figure 2).

**Table 2:** : Models investigating the temporal variation of colonisation (γ), extinction (ε) and detection probability (p).

Model	no. Par.	-2loglike	AICc	ΔAICc
ψ, γ(.) , ε(time), p(time)	18	2907.01	2945.54	0
ψ, γ (time), ε (time), p(time)	19	2906.96	2947.79	2.24
ψ, γ (.) , ε (.) , p(time)	17	2912.81	2949.07	3.52
ψ, γ (time), ε (.) , p(time)	18	2912.81	2951.34	5.8
ψ, γ (.) , ε (.) , p(.)	15	2940.15	2971.91	26.36

Models used for individual parameters were γ (size + matrix), ε (size + composition + veg + matrix), p (size + composition + matrix) and ψ (size + matrix). The models were ranked by AICc values. (.) = parameter is constant over time, only covariates included, (time) = parameter varies between years, covariates and intercepts for each year included.

### 3.2: Internal validation

For the cross-validation data-sets used to train the models the optimized thresholds varied between 0.42 and 0.57, highlighting the differences in prevalence of presences and absences in the different data-sets. The mean of the correct classification rate decreased from 0.70 (+/- 0.07) for the 2006 data to 0.65 (+/- 0.06) for the 2007 data and 0.64 (+/- 0.10) for the 2008 data. Overall the correct classification rate ranged from 0.54 to 0.74 with an overall mean of 0.70 (+/- 0.07). The cross-validation procedure for the top model estimated an overall mean AUC of 0.74 (+/- 0.06) which indicates “satisfying to good” predictive abilities [68]. The AUC of the model stayed relatively constant for grand skink occupancy predictions for all three years. The mean TSS was 0.38 (+/- 0.13) suggesting a predictive ability of the model better than random. Similar to the correct classification rate the TSS revealed a better predictive ability of the first year [mean TSS 2006: = 0.38 (+/- 0.13) compared to the following two years (mean TSS 2007: 0.27 (+/- 0.14), 2008: 0.29 (+/-0.18), Appendix S2] and showed an increase in the associated standard error.

### 3.3: External validation

The naïve occupancy rates for the 67 habitat patches in tussock grasslands and 64 habitat patches in pasture grasslands monitored in 2010 were 0.55 and 0.44, respectively, with an overall mean naïve occupancy rate of 0.49. Using the logistic regression function estimated by the top-ranked model the average occupancy probability for habitat patches in tussock grasslands was 0.50 (+/-0.20) and for habitat patches in pasture grasslands was 0.24 (+/- 0.15) with an overall mean occupancy probability of 0.38 (+/- 0.21). Using an optimised threshold of 0.43 derived from the 2006 data-set, a TSS of 0.49 was estimated for the model using the total 2010 data-set. The AUC of the overall model was estimated as 0.76 when using the total 2010 data-set. The TSS and AUC measures indicate that the model is a good predictor of grand skink occupancy when combining data for both matrix types.

Assessing the predictive ability of the top-ranked model separately for both matrix types resulted in higher TSS and AUC values for habitat patches in tussock grasslands. The model predictions for presences were more accurate for habitat patches in tussock grasslands (sensitivity = 0.76) compared to habitat patches in pasture grasslands (sensitivity = 0.43). For both, habitat patches in tussock and pasture grasslands, the specificity (correctly identified absences) measures were higher than the sensitivity measures (Appendix S3).

## Discussion

### 4.1: Habitat patch-specific metapopulation parameters

The strength and direction of the influence of habitat patch characteristics differed between the metapopulation parameters. The size of the habitat patch had the most influence on all metapopulation parameters. In our study larger habitat patches had higher occupancy and colonisation probabilities and lower extinction probabilities. Many empirical and theoretical studies have found this relationship [9,10]. By definition, local populations in habitat patches are small and thus prone to extinction. Large habitat patches, however, can accommodate larger populations and provide more resources such as food and refugia making a population less prone to extinction due to stochastic processes [9,69]. Furthermore, the size of the habitat patch influences colonisation probabilities by increasing the chance of an animal encountering a habitat patch while moving through the landscape [9]. In our study, the size of the habitat patch was positively correlated with the height of the outcrop (KG, unpublished data). In her study on the homing abilities of grand skinks, Stanley [70] found that grand skinks were more likely to return to their home-outcrop if it was visible from the release location (and within 70m). Therefore grand skinks may be more likely to colonise a habitat patch which is visible from their residential habitat patch.

Matrix type was as important as habitat patch size for grand skink metapopulation processes. Habitat patches in native tussock grassland had higher occupancy and colonisation probabilities and lower extinction probabilities than habitat patches in modified pasture grasslands, similar to the findings of Seddon et al. [45]. Higher colonisation probabilities indicate higher rates of successful movements of grand skinks between habitat patches in tussock grasslands. This confirms the findings of Berry et al. [38], who showed that populations of grand skinks on habitat patches in pasture were genetically more isolated than grand skinks in habitat patches in tussock grasslands. In contrast to tall tussock grasses, close-cropped pasture grasslands do not provide shelter during movements of grand skinks between habitat patches. Higher perceived predation risk [71]could reduce movements of grand skinks between habitat patches and therefore colonisation probabilities. A study using radio-transmitters on grand skinks recorded more movements between habitat patches in tussock grasslands than in pasture grasslands (KG, unpublished data). Additionally matrix type was more important for colonisation probabilities than was distance between habitat patches, indicating that the reluctance of grand skinks to cross pasture grasslands had a greater effect on the reduction of movements between habitat patches than did the ability of the grand skink to travel the distance.

Matrix type also influenced extinction probabilities. Habitat-patches surrounded by tussock grasslands had lower extinction probabilities than for habitat patches embedded in pasture grassland. Outcrops in structurally more diverse tussock grasslands might provide a better invertebrate food source for grand skinks, thus leading to reduced extinction rates. Tocher et al. [36] found higher invertebrate abundances but similar species composition in pasture habitats compared to tussock habitats, which suggests that differences in food resources supplied by the matrix are not influencing extinction probabilities. A more likely explanation is a higher level of predation of grand skinks in pasture habitats because of increased predator numbers caused by relatively high rabbit abundance [72]. Furthermore, the reluctance of grand skinks to move between habitat patches in pasture grassland limits possible rescue effects. Together increased predation risk and a decreased disposition to move across pasture grasslands lead to higher extinction probabilities for grand skink populations in habitat patches in a pasture grassland matrix.

Overall, the final model predicted higher occupancy probabilities of habitat patches in tussock grasslands compared to pasture grasslands which were also found for generic (not patch specific) rates by Whitaker [37] and Seddon et al. [45]. Higher occupancy in tussock results from a combination of higher colonisation and lower extinction probabilities than for habitat patches in pasture grasslands.

The composition of habitat patches had no great influence on occupancy and colonisation probabilities and therefore composition was not included in the models of occupancy and colonisation probabilities. However, composition influenced extinction probabilities which were higher in habitat patches consisting of dispersed rock-tors compared to habitat patches with one discrete rock-tor. Although dispersed rock-tors could provide more cracks and crevices as refugia for grand skinks, thereby potentially reducing predation rates and providing space for greater abundances of grand skinks, rock-cracks might be too shallow to provide shelter especially in winter. Murphy [41] reported differences in crevice dimension choices of adult, sub-adult and juvenile grand skinks, with adult skinks preferring much larger crevices than those selected by juvenile animals. Additionally, the tendency of the highly saxicolous grand skinks to stay on rock-tors and not to move through grasslands would reduce interaction of animals on dispersed rock-tors and therefore mating opportunities and reproduction in those outcrops, increasing extinction probabilities.

Vegetation cover was retained only in the extinction probability model. The more vegetation on a habitat patch the lower the extinction probability for that specific habitat patch. Vegetation cover on an outcrop plays an important role in providing food for grand skinks directly in the form of berries and indirectly by attracting and housing invertebrates [36], especially as outcrops support vegetation which is largely absent from the matrix. Grand skinks also use the vegetation as refugia from predators and for shade against overheating in hot temperatures. Colonisation and occupancy models did not include vegetation cover indicating that the amount of vegetation cover might not contribute to a grand skinks decision to colonise a habitat patch and might not be essential for occupancy by grand skinks. However, Whitaker [37] found that occupancy of grand skinks was correlated with the presences of fruiting shrubs. The categorization of vegetation cover chosen in this study was very broad (to limit time for data collection and observer training), and more detailed information on the composition of the vegetation could improve our understanding of its role in metapopulation dynamics of grand skinks.

Distance to the nearest habitat patch was not included in the final models for occupancy or colonisation probabilities. This measure has been included in metapopulation studies before to incorporate the ability of the individuals to travel the distances between habitat patches for colonisation events [4]. Although adult grand skinks have been found to move mostly between 0-50 m ( [40], KG, unpublished data), and therefore indicating that larger distances between habitat patches could reduce colonisation probabilities, juvenile grand skinks have been reported to move between 100 and 200m [48], which is further than distances between habitat patches in our study. This could lead to the low influence of distance between habitat patches in the colonisation model. Also, distance between habitat patches has been criticised for being an overly simplistic measure in the literature before [7,49], and a more sensitive measures might provide better information. Factors leading to animals leaving their habitat patch are various, high population density in the current patch [73] and perceptual range [74]. Furthermore the speed, direction and linearity of movements between habitat patches are dependent on many factors, e.g. the matrix between the habitat patches, predator densities and terrain [21]. With more detailed information on grand skink movements between habitat patches more sensitive isolation measures could be developed for future analysis.

We found temporal variation in extinction and detection probabilities, whereas colonisation probabilities remained constant over time. Seddon et al. [45] also found detection probability to vary between years, however, contrary to our results their models suggested constant extinction probabilities and colonisation probabilities to vary between years. Their study encompassed five years (compared to 3 years for this study) which would potentially provide a more robust estimation of metapopulation parameters. However, they did not derive patch-specific rates that take outcrop size, composition, vegetation cover and isolation into consideration. Occupancy probabilities in our study increased from 2006 to 2007 because of lower extinction probabilities than colonisation probabilities. An increase in extinction probabilities for the period between 2007/8 to the same level as the colonisation probabilities resulted in similar occupancy in 2007 and 2008. This and the results by Seddon et al. [45] indicate that there are likely to be fluctuations in colonisation and extinction probabilities in the grand skink populations over longer time frames and long-term monitoring will be required to determine the metapopulation trends. Fluctuations in extinction and colonisation probabilities could be caused by fluctuating predator population sizes between years. Furthermore, climatic factors directly influence skink behaviour and could cause colonistaion variability [40,41,48]. Also, variation in reproduction success due to climatic and demographic factors, especially for species with low reproductive outputs such as the grand skink [75] could lead to variability in the metapopulation parameters. Our study shows no overall decline in grand skink occupancy at this study site supporting the findings of Seddon et al. [45].

### 4.2: Habitat patch-specific detection probabilities

The average detection probabilities of 0.53-0.60 in our study are lower than in previous studies of grand skinks [45]. Although we conducted surveys in weather conditions chosen to ensure high skink activity it is likely that for heliotherm animals such as skinks detection probabilities will differ with small changes in weather conditions. This and the abilities of an observer to detect a skink lead to the differences of the detection probabilities between years and between studies, emphasizing the need to account for incomplete detection probabilities in the study design.

Detection probabilities were higher in larger habitat patches which are likely to accommodate a larger number of animals. With increasing abundance of grand skinks the likelihood of detecting an individual during the 5-min survey probably also increases. Furthermore detection probabilities of grand skinks were higher in habitat patches surrounded by tussock grasslands compared to pasture grasslands. These results contrast with the findings of Seddon et al. [45] who found no differences between the detection probabilities of the two matrix types, but confirm the findings by Whitaker [37]. Whitaker [37] additionally reported higher densities of grand skinks in habitat patches surrounded by tussock rather than pasture habitat and a higher proportion of single animals in habitat patches surrounded by pasture grassland. A higher density of grand skinks in habitat patches in tussock grasslands would make it more likely for observers to see at least one skink during surveys.

### 4.3: Validation process

The internal and external validation process showed that our model had adequate predictive properties, but correct-classification-rates decreased over the three year study-period. The validation process also revealed that the model predicted occupancy of grand skinks in pasture grasslands less accurately than in tussock grasslands. Fielding and Bell [33] emphasized that sometimes species do not occupy all the available habitat, and this will influence the ability of the model to discriminate between positive and negative locations. For example, higher predation pressure in pasture grasslands could result in some suitable outcrops not being inhabited by grand skinks. If predation events on outcrops, and resulting extinction of a grand skink population, are random and cannot be attributed to a specific habitat patch characteristic that is included in the model, the resulting model will have a lower predictive ability. Another possible explanation could be that there are factors influencing the population dynamics in pasture grasslands in differently than in tussock grassland, and that those factors were not measured in this study.

Results for predictive abilities from our external validation process were similar to the internal validation process. Because of the restricted range of grand skinks the study sites for the external validation process were still relatively close to the original study sites. Therefore, bias potentially introduced by spatial autocorrelation could be similar to that of internal evaluation procedures. However, our external validation data were collected several years after the original data-set, introducing temporal independence.

To our knowledge there are no evaluation measures available in the literature that take into consideration the incomplete detectability accounted for in our model. Evaluation measures for dichotomous data are based on comparing correctly and incorrectly predicted cases; however as a consequence of acknowledging incomplete detectability in the modelling process, we can no longer assume that all absences observed are true absences. Because of the incomplete detectability of the species the model will predict a number of observed absences as presences which common evaluation measures will penalise as wrong predictions. Owing to our study design with four surveys per monitoring period only 3-6% of observed absences would be classified as presences. All presences would be classified correctly by a ‘perfect’ model because of the underlying assumption of correct identification of the species. By not accounting for incomplete detectability specificity values in the evaluation measures would be wrong by 3-6% in our study. Future development of analytical tools that incorporate incomplete detectability in models validation will be needed to address this problem.

Our study emphasized that metapopulation dynamics are not driven by habitat patch size and isolation alone, but also by other attributes of the habitat patch that influence the ecology of the study species and by the surrounding matrix. For example, in our study, matrix type was a better measure of habitat patch isolation than distance. The disparity between various studies in the relative importance of habitat patch and matrix are likely due to differences in the ecology of the study species and the data collection methods. Because of this variation it is imperative to use existing knowledge of the ecology of the study species to decide which variables play important roles in the metapopulation dynamics. We chose biologically relevant and easy-to-measure variables so the key data could be collected relatively quickly and with low cost, addressing important ecological factors influencing the metapopulation dynamics of grand skinks. The results of this study will enable the classification of habitat patch suitability for grand skinks and guide planning for translocations and the creation and management of skink conservation areas. In addition, shortcomings of classic evaluation measures for models incorporating detection probabilities have been identified for future research.

## Supporting Information

Appendix S1 **Model-sets developed to identify the influence of habitat patch characteristics on metapopulation parameters.**
(DOC)

Appendix S2 **True skill statistic (TSS) over the whole range of possible sensitivity and specificity values.**
(PDF)

Appendix S3 **Accuracy measures for predictive abilities of the final model on the independent data-set of 2010.**
(DOC)
