# Supplementary material for: Matrix Matters: Differences of Grand Skink Metapopulation Parameters in Native Tussock Grasslands and Exotic Pasture Grasslands
Source: PLoS One. 2013 Oct 2;8(10):e76076. doi: 10.1371/journal.pone.0076076 (PMC3788794; doi:10.1371/journal.pone.0076076)
Supplement: Appendix S1 — Model-sets developed to identify the influence of habitat patch characteristics on metapopulation parameters. (DOC) [file pone.0076076.s001.doc]

**Appendix S1.** Model-sets developed to identify the influence of habitat patch-characteristics on the metapopulation parameters occupancy (ψ), colonisation (γ), extinction (ε) and detection probability (p). The highest ranked model (ΔAICc = 0.00) for each metapopulation parameter is marked bold.

|  | Habitat patch characteristics | | | | | ΔAICc values (Akaike weights) of probability models | | | |
| --- | --- | --- | --- | --- | --- | --- | --- | --- | --- |
| Model | Matrix type^a^ | Size^b^ | Compo-sition^c^ | Vegetation cover^d^ | Isolation^e^ | ψ | γ | ε | p |
| 1 | x |  |  |  |  | 34.84  (0.00) | 11.77  (0.00) | 11.30  (0.00) | 64.87  (0.00) |
| 2 | x | x |  |  |  | **0.00**  **(0.22)** | **0.00**  **(0.35)** | 7.22  (0.01) | 25.26  (0.00) |
| 3 | x |  | x |  |  | 36.64  (0.00) | 12.81  (0.00) | 8.94  (0.00) | 53.36  (0.00) |
| 4 | x |  |  | x |  | 36.26  (0.00) | 13.77  (0.00) | 11.26  (0.00) | 65.09  (0.00) |
| 5 | x | x | x |  |  | 0.08  (0.21) | 1.82  (0.14) | 1.39  (0.18) | **0.00**  **(0.55)** |
| 6 | x |  | x | x |  | 38.01  (0.00) | 14.86  (0.00) | 7.44  (0.01) | 53.25  (0.00) |
| 7 | x | x |  | x |  | 1.87  (0.09) | 1.44  (0.17) | 7.86  (0.01) | 26.12  (0.00) |
| 8 | x | x | x | x |  | 2.03  (0.08) | 3.25  (0.07) | **0.00**  **(0.36)** | 0.48  (0.43) |
| 9 |  | x |  |  |  | 22.92  (0.00) | 22.76  (0.00) | 7.01  (0.01) | 30.37  (0.00) |
| 10 |  | x | x |  |  | 23.39  (0.00) | 24.28  (0.00) | 2.53  (0.10) | 8.15  (0.01) |
| 11 |  | x |  | x |  | 24.98  (0.00) | 24.82  (0.00) | 7.11  (0.01) | 30.45  (0.00) |
| 12 |  | x | x | x |  | 25.43  (0.00) | 26.32  (0.00) | 0.37  (0.30) | 7.57  (0.01) |
| 13 |  |  | x |  |  | 54.63  (0.00) | 27.30  (0.00) | 9.33  (0.00) | 60.77  (0.00) |
| 14 |  |  | x | x |  | 54.84  (0.00) | 29.25  (0.00) | 7.35  (0.01) | 59.34  (0.00) |
| 15 |  |  |  | x |  | 53.05  (0.00) | 28.16  (0.00) | 10.86  (0.00) | 69.83  (0.00) |
| 16 | x |  |  |  | x | 36.87  (0.00) | 13.82  (0.00) | - | - |
| 17 | x | x |  |  | x | 0.78  (0.15) | 1.93  (0.13) | - | - |
| 18 | x |  | x |  | x | 38.68  (0.00) | 14.86  (0.00) | - | - |
| 19 | x |  |  | x | x | 38.29  (0.00) | 15.82  (0.00) | - | - |
| 20 | x | x | x |  | x | 0.89  (0.14) | 3.76  (0.05) | - | - |
| 21 | x |  | x | x | x | 40.06  (0.00) | 16.93  (0.00) | - | - |
| 22 | x | x |  | x | x | 2.63  (0.06) | 3.42  (0.06) | - | - |
| 23 | x | x | x | x | x | 2.82  (0.05) | 5.24  (0.03) | - | - |
| 24 |  | x |  |  | x | 24.16  (0.00) | 24.85  (0.00) | - | - |
| 25 |  | x | x |  | x | 24.64  (0.00) | 26.38  (0.00) | - | - |
| 26 |  | x |  | x | x | 26.23  (0.00) | 26.92  (0.00) | - | - |
| 27 |  | x | x | x | x | 26.71  (0.00) | 28.44  (0.00) | - | - |
| 28 |  |  | x |  | x | 56.70  (0.00) | 29.39  (0.00) | - | - |
| 29 |  |  | x | x | x | 56.91  (0.00) | 31.35  (0.00) | - | - |
| 30 |  |  |  | x | x | 55.11  (0.00) | 30.25  (0.00) | - | - |
| 31 |  |  |  |  | x | 54.80  (0.00) | 28.41  (0.00) | - | - |

AICc – second-order bias corrected Akaike Information Criterion

x – variable included in the model

^a^ categorical variable (pasture or tussock grassland)

^b^ categorical variable (small, medium or large)

^c^ categorical variable (discrete or clustered)

^d^ categorical variable (none, moderate or abundant)

^e^ continuous variable ( nearest neighbour distance [m])
