# Supplementary material for: Matrix Matters: Differences of Grand Skink Metapopulation Parameters in Native Tussock Grasslands and Exotic Pasture Grasslands
Source: PLoS One. 2013 Oct 2;8(10):e76076. doi: 10.1371/journal.pone.0076076 (PMC3788794; doi:10.1371/journal.pone.0076076)
Supplement: Appendix S2 — True skill statistic (TSS) over the whole range of possible sensitivity and specificity values. (PDF) [file pone.0076076.s002.pdf]

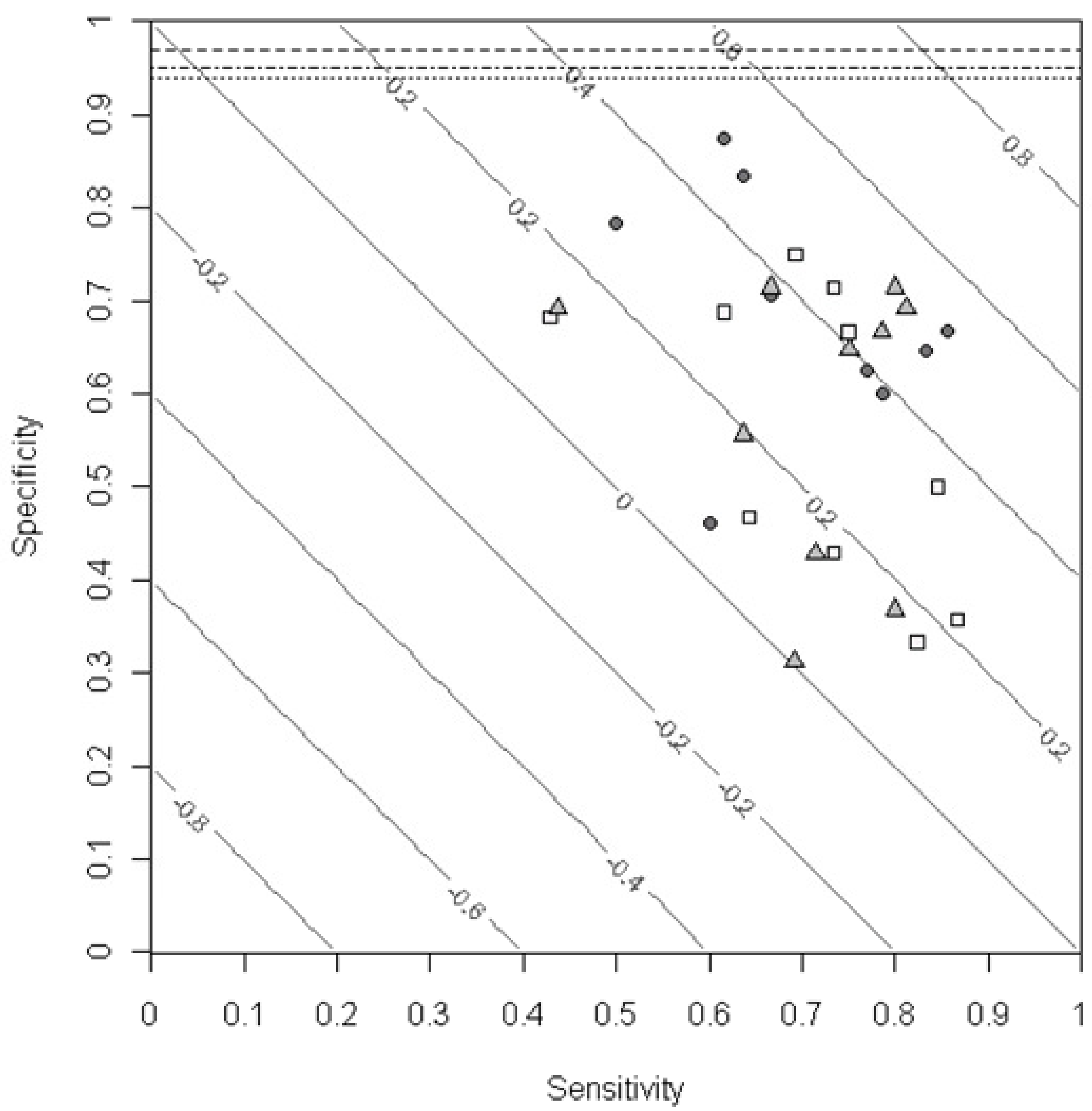

Appendix S2: True skill statistic (TSS) over the whole range of possible sensitivity and specificity values. TSS values (grey contours) of -1 indicate predictive abilities of not better than a random model, 0 indicates an indiscriminate model and +1 a perfect model. TSS for the 10-fold cross-validation procedure are marked as circles for year 2006, squares for year 2007 and triangles for year 2008. The horizontal lines represent maximum possible values of specificity due to varying detection probabilities per year (dashed line: 2006, dotted line: 2007, dashes and dots: 2008)
