# Supplementary material for: Matrix Matters: Differences of Grand Skink Metapopulation Parameters in Native Tussock Grasslands and Exotic Pasture Grasslands
Source: PLoS One. 2013 Oct 2;8(10):e76076. doi: 10.1371/journal.pone.0076076 (PMC3788794; doi:10.1371/journal.pone.0076076)
Supplement: Appendix S3 — Accuracy measures for predictive abilities of the final model on the independent data-set of 2010. (DOC) [file pone.0076076.s003.doc]

**Appendix S3**: Accuracy measures for predictive abilities of the final model on the independent data-set of the year 2010. The threshold dependent measures were estimated using the optimised threshold of 0.43 which was derived from the year 2006 data-set which was used to train the occupancy model. The true skill statistic (TSS) ranges from -1 indicating predictions no better than random to +1 for perfect predictive abilities. The AUC ranges from 0.5 for an indiscriminate model to +1 for perfect predictive abilities.

|  | correct classification rate | sensitivity | specificity | TSS | AUC |
| --- | --- | --- | --- | --- | --- |
| Pasture 2010 | 0.70 | 0.43 | 0.92 | 0.36 | 0.71 |
| Tussock 2010 | 0.76 | 0.76 | 0.83 | 0.59 | 0.84 |
| total | 0.75 | 0.62 | 0.87 | 0.49 | 0.76 |
